# Anti-angiogenic therapeutic strategies in hereditary hemorrhagic telangiectasia

**DOI:** 10.3389/fgene.2015.00035

**Published:** 2015-02-11

**Authors:** Daniela S. Ardelean, Michelle Letarte

**Affiliations:** ^1^Department of Pediatrics, The Hospital for Sick ChildrenToronto, ON, Canada; ^2^Molecular Structure and Function Program, Peter Gilgan Centre for Research and Learning, The Hospital for Sick ChildrenToronto, ON, Canada; ^3^Department of Immunology, University of TorontoToronto, ON, Canada; ^4^Heart and Stroke Richard Lewar Centre of Excellence, University of TorontoToronto, ON, Canada

**Keywords:** angiogenesis, HHT, endoglin, Alk1, VEGF, anti-angiogenic therapy, anti-VEGF, inflammation

## Abstract

Hereditary hemorrhagic telangiectasia (HHT) is an autosomal dominant vascular dysplastic disorder, characterized by recurrent nosebleeds (epistaxis), multiple telangiectases and arteriovenous malformations (AVMs) in major organs. Mutations in *Endoglin* (*ENG* or *CD105*) and *Activin receptor-like kinase 1* (*ACVRL1* or *ALK1*) genes of the TGF-β superfamily receptors are responsible for HHT1 and HHT2 respectively and account for the majority of HHT cases. Haploinsufficiency in *ENG* and *ALK1* is recognized at the underlying cause of HHT. However, the mechanisms responsible for the predisposition to and generation of AVMs, the hallmark of this disease, are poorly understood. Recent data suggest that dysregulated angiogenesis contributes to the pathogenesis of HHT and that the vascular endothelial growth factor, VEGF, may be implicated in this disease, by modulating the angiogenic–angiostatic balance in the affected tissues. Hence, anti-angiogenic therapies that target the abnormal vessels and restore the angiogenic–angiostatic balance are candidates for treatment of HHT. Here we review the experimental evidence for dysregulated angiogenesis in HHT, the anti-angiogenic therapeutic strategies used in animal models and some patients with HHT and the potential benefit of the anti-angiogenic treatment for ameliorating this severe, progressive vascular disease.

## HHT IS A SEVERE SYSTEMIC VASCULAR DYSPLASTIC DISEASE

Hereditary hemorrhagic telangiectasia (HHT) is an inherited systemic vascular dysplastic disorder, associated with multiple mucocutaneous telangiectases, recurrent nasal and gastrointestinal bleeding episodes and large arteriovenous malformations (AVMs) in lungs, liver and brain. More than 80% of HHT patients carry a mutation in *Endoglin* (*ENG;* HHT1) or *Activin receptor-like kinase 1* (*ACVRL1;* HHT2) genes that code for receptors of the transforming growth factor β (TGF-β) superfamily. Reduced expression of *ENG* and *ACVRL1* (haploinsufficiency) leads to a similar HHT phenotype. However, there is a higher incidence of pulmonary and cerebral AVMs in HHT1, while hepatic AVMs and gastrointestinal telangiectases are more often diagnosed in HHT2 patients (Friesel et al., 1987; Govani and Shovlin, 2009; Storch and Hoeger, 2010). This suggests that the HHT phenotype is influenced by the tissue distribution and function of *ENG* and *ACVRL1.* Although pulmonary arterial hypertension (PAH) is a much rarer event than the occurrence of HHT, it has also been associated with *ACVRL1* and *ENG* mutations (Govani and Shovlin, 2009). In these cases, PAH likely results from a dysfunctional relationship between *ACVRL1, ENG* and the bone morphogenic protein receptor type II, BMPR2, another member of the TGF-β superfamily of receptors, whose mutations are often the cause of inherited as well as sporadic cases of PAH (Atkinson et al., 2002). Despite extensive work in HHT, no cure for this disease exists. Symptomatic treatments, including AVM embolization, offer some relief, yet HHT is a progressive, severe and potentially life-threatening disease. Recent experimental data and some clinical studies suggest that anti-angiogenic therapies targeting the abnormal vasculature may have some benefits in HHT.

## DYSREGULATED ANGIOGENESIS IN HHT

In HHT, the mechanisms leading to predisposition and formation of AVMs, the direct connections between arteries and veins, are yet to be determined. One proposed mechanism is defective arteriovenous differentiation, observed in *Eng* and *Alk1* null embryos that develop AVMs (Oh et al., 2000; Sorensen et al., 2003), but absent in the endothelial-targeted *Eng* (*Eng*-iKO^e^) and *Alk1* inducible knockout (*Alk1*-iKO^e^) mice (Tual-Chalot et al., 2014). Moreover, focal regression of capillaries leading to formation of AVMs has also been postulated in HHT, however, supporting data for this model are still lacking (Lopez-Novoa and Bernabeu, 2010). Recently, it was shown that wound injury was necessary for development of AVMs in adult *Alk1*-iKO^e^ (Park et al., 2009) and *Eng*1-iKO^e^ mice (Garrido-Martin et al., 2014). In addition, intracerebral injection of an adenovirus expressing VEGF contributed to pathogenesis of cerebral AVMs in several transgenic *Eng* KO mice (Choi et al., 2014). Interestingly, AVMs were found more often at the site of vascular injury or turbulent flow, indicating that these local vascular changes may precipitate the development of AVMs.

**FIGURE 1:**
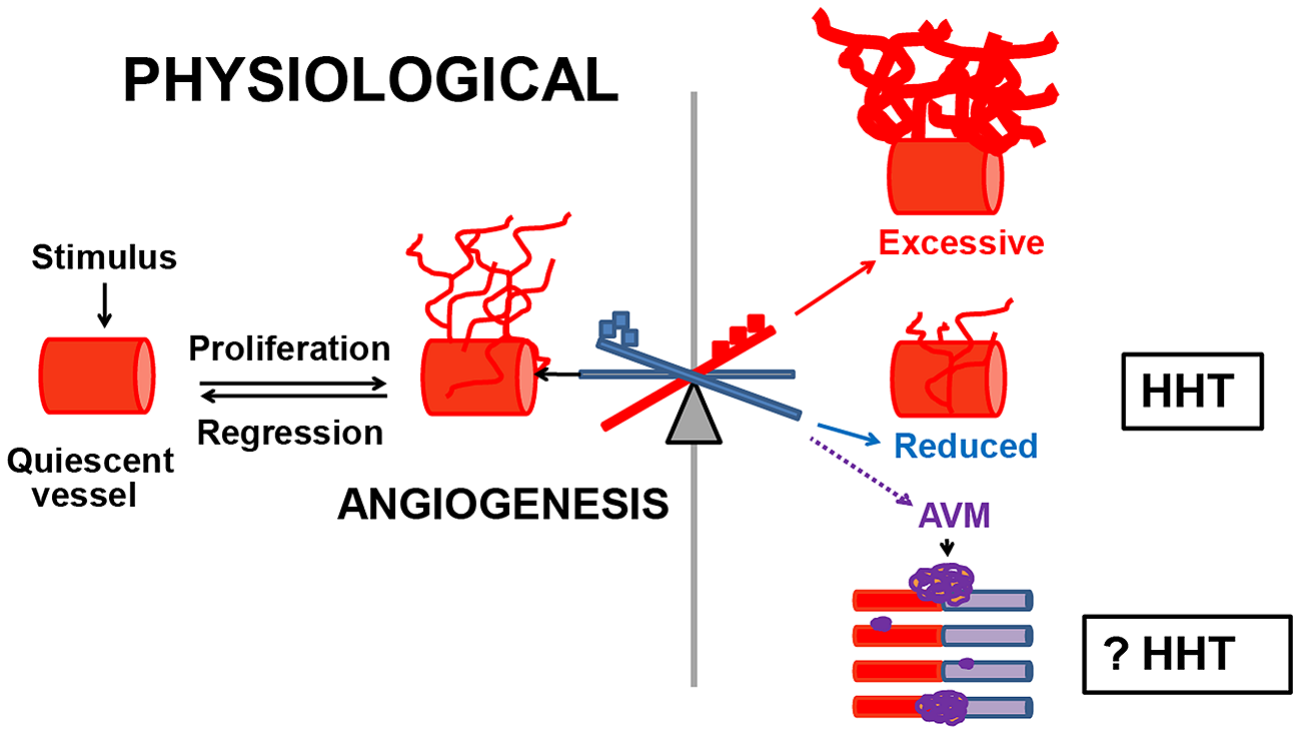
**Dysregulated angiogenesis in HHT.** Normally, when quiescent vessels are activated, they proliferate, migrate and mature until the physiological needs of the organ have been met, and then regress. Pathological angiogenesis commences when the angiogenic–angiostatic balance is disrupted, leading to dysregulated angiogenesis, excessive or reduced tissue MVD and possibly to AVMs. In *Eng^+/-^* and *Alk1^+/-^* models of HHT, an imbalance in the pulmonary angiostatic TSP-1 and vascular destabilizing factor Ang-2 respectively, led to reduced peripheral lung MVD in both models. However, it is unknown if dysregulated angiogenesis is involved in the development of AVMs in HHT. 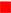 Angiogenic factors; 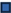 angiostatic proteins.

We demonstrated that dysregulated angiogenesis occurs in *Eng^+/-^* and *Alk1^+/-^* mouse models of HHT. Angiogenesis is the *de novo* formation of vessels from the pre-existent vascular tree, in response to a stimulus. This biological process is controlled by pro-angiogenic factors that promote vascular growth and angiostatic factors that induce vascular regression. Physiological angiogenesis occurs during development and in healthy individuals, in wound injury and repair, menstruation, pregnancy (Reisinger et al., 2007), and in testis (Collin and Bergh, 1996) and hair follicles (Yano et al., 2003). Under normal conditions, angiogenesis is short-lived, due to finely tuned regulatory mechanisms. In contrast, pathological angiogenesis is abnormal, persists indefinitely and leads to excessive or insufficient generation of new vessels and likely to abnormal ones such as AVMs (**Figure 1**). Pathological angiogenesis contributes to disease progression in cancer (Nagy et al., 2010), chronic inflammatory and chronic infectious diseases (Carmeliet, 2003). However, the possibility that pathological angiogenesis occurs in HHT has not been explored previously. A few clinical studies showed that vascular endothelial growth factor VEGF, a major angiogenic protein, was elevated in circulation and tissues in HHT patients (Sadick et al., 2005a,b). In addition, it was reported that *Alk1*^+/^*^-^* mice had higher VEGF mRNA and protein levels in lungs, liver and intestine, when compared to wild type (WT) mice (Shao et al., 2009). These data suggested that VEGF might play a pathogenic role in HHT and that targeting VEGF in animal models of HHT and patients could be beneficial.

Pathological angiogenesis can be evaluated in experimental models by several approaches, from measuring the levels of angiostatic and angiogenic factors in tissues and circulation to *in vitro* and *in vivo* quantification of tissue microvessel density (MVD). To investigate if pathological angiogenesis occurs in HHT, we used the adult *Eng^+/-^* and *Alk1^+/-^* mice on the C57BL/6 background. *Eng^+/-^* and *Alk1^+/-^* mice lack AVMs, but develop pulmonary peripheral vascular rarefaction, associated with right ventricular hypertrophy (RVH) and spontaneous PAH (Toporsian et al., 2010; Jerkic et al., 2011). Recently, we demonstrated that the lungs are the only organ showing pathological angiogenesis or reduced MVD in both *Eng^+/-^* and *Alk1^+/-^* mice (Ardelean et al., 2014a). The reduction in peripheral lung MVD was associated with a genotype-specific molecular angiogenic–angiostatic imbalance. *Eng*^+/^*^-^* lungs had a fourfold increase in the angiostatic factor thrombospondin-1 (TSP-1), which was inversely correlated with the endoglin levels. This finding was confirmed *in vitro* in endoglin-deficient endothelial cells. In contrast, *Alk1^+/-^* lungs showed an augmentation in the vascular destabilizing protein Ang-2, whereas the tissue TSP-1 was normal. However, in both *Eng*^+/^*^-^* and *Alk1^+/-^* lungs, the levels of the major pro-angiogenic and vascular regulator VEGF were unchanged when compared to WT mice, suggesting that factors other than VEGF are implicated in modulation of the tissue angiogenic–angiostatic balance in these mouse models of HHT. These data indicate that an impairment in the molecular balance of angiogenic and angiostatic factors can lead to changes in tissue MVD, in disorders of dysregulated angiogenesis, such as HHT (**Figure 1**).

Furthermore, a 70% increase in TSP-1 levels was also noted in the liver of *Eng*^+/^*^-^* mice versus WT littermates, whereas the VEGF levels were normal. This hepatic imbalance in the TSP-1/VEGF levels was not accompanied by an alteration in liver MVD in *Eng*^+/^*^-^* mice, indicating that a critical molecular threshold must be reached in the levels of angiostatic versus pro-angiogenic factors in order to affect tissue MVD. The liver of *Alk1^+/-^* mice had normal MVD and tissue levels of Ang-2 and VEGF.

Surprisingly, even though VEGF does not appear to play a major pathogenic role in *Eng*^+/^*^-^* and *Alk1^+/-^* mice, blocking VEGF with the monoclonal antibody G6-31 led to improvement in the molecular and microvascular phenotype in the affected lungs of these mice (Ardelean et al., 2014a). Anti-VEGF treatment normalized the pulmonary TSP-1 and Ang-2 levels in *Eng*^+/^*^-^* and *Alk1^+/-^* mice respectively, increased and restored the lung peripheral MVD and alleviated the RVH in the treated mice. This suggests that VEGF may be a critical regulator of multiple angiogenic pathways in HHT. Thus, we speculate that the anti-VEGF treatment, by decreasing the pulmonary VEGF levels in both *Eng*^+/^*^-^* and *Alk1^+/-^* mice, reduced the tissue TSP-1 and Ang-2 levels respectively, either (a) directly or (b) possibly via p38 MAPK, a protein involved in signaling transduction for VEGF, some of the TGF-β superfamily of ligands, TSP-1 and Ang-2. However, these currently unknown mechanisms need to be investigated.

In support of the concept that VEGF is a critical angiogenic regulator, G6-31 treatment also led to prevention and reversal of mucocutaneous AVMs in *Alk1*-iKO^e^ mouse model of HHT (Han et al., 2014), indicating that a reduction in VEGF levels is able to inhibit the growth of AVMs. Thus, anti-VEGF treatment restored the tissue TSP-1 levels and improved the pulmonary microvascular phenotype in *Eng*^+/^*^-^* and *Alk1^+/-^* mice, while it prevented and halted the generation of AVMs in the *Alk1* iKO^e^ model of HHT.

## ANTI-ANGIOGENIC THERAPEUTIC STRATEGIES IN HHT

Judath Folkman, the pioneer of angiogenesis and anti-angiogenic therapy, proposed that angiogenesis can be blocked directly, using drugs that alter EC migration and proliferation or indirectly, via modulation of growth factors that act on the vascular endothelium (Folkman and Klagsbrun, 1987). As HHT is a systemic vascular dysplasia of dysregulated angiogenesis, medication that targets directly or indirectly the abnormal vessels could have beneficial effects in this disease. For example, anti-VEGF treatment and thalidomide that have been tested in HHT exert direct anti-angiogenic properties. Propranolol and timolol are in the process of being tested in HHT and have mostly indirect anti-angiogenic effects. Tacrolimus, interferon (IFN)-α and infliximab, used only in isolated cases of HHT, are predominantly immunomodulatory drugs with some anti-angiogenic capabilities (**Table 1**). Here, the role of immune system in pathogenesis of HHT is mostly unknown. Whether dysfunctional immune cells contribute directly or indirectly to pathological angiogenesis and whether immunomodulatory therapies could be beneficial in patients diagnosed with HHT, need to be determined.

**Table 1:** Mechanisms of several anti-angiogenic and immunomodulatory therapies in HHT.

Therapy	Angiogenic effects	Evidence	Immuno-modulator	Evidence
**Tested in patients with HHT**
Anti-VEGF (G6-31 murine monoclonal antibody)	**+++**	↑ MVD, ↓ TSP-1, and ↓ Ang-2 in lungs of *Eng^+/-^* and *Alk1^+/-^* mice, respectively (Ardelean et al., 2014a) ↓ vessel area and density in the skin of *Alk1* iKO mouse (Han et al., 2014)	**+**	↓ inflammation in the colon of DSS-induced *Eng^+/-^* colitis (Ardelean et al., 2014b) and the skin of *JunB* and *c-Jun* iKO mice (Schonthaler et al., 2009)


Thalidomide	**++**	**High dose (200 mg/kg)** ↓ vascular area in rabbit cornea (D’Amato et al., 1994) ↓ VEGF, migration and tube formation in EA.hy 926 EC (Komorowski et al., 2006) ↓ VEGFR2 and neuropilin-1 in zebrafish embryos (Yabu et al., 2005) **Low dose (75 mg/kg)** ↑ pericyte recruitment in neonatal mouse retina (Lebrin et al., 2010)	**++**	↓ human monocyte-dependent secretion of several cytokines ↑ proliferation and function of circulating human T lymphocytes ↓ number of adhesion molecules in HUVEC ↑ number of NK cells in human multiple myeloma cell lines (Teo, 2005)
**To be tested in HHT patients**
Propranolol	++	↓ proliferation and ↑ apoptosis in HUVEC (Xie et al., 2013) ↓ VEGF and MMP-9 in hemangioma-derived EC (Storch and Hoeger, 2010) ↓ endoglin, ACVRL1, PAI-1 in HMEC-1 and HUVEC (Albinana et al., 2012)	**+**	↑ number of T cells, ↓ NK cell activity in human blood (Maisel et al., 1991)
**Not yet proposed for HHT patients**
Tacrolimus	**+**	↓ VEGF-induced mouse EC tube formation (Siamakpour-Reihani et al., 2011) ↑ endoglin and *Alk1* in HUVEC (Albinana et al., 2011)	**+++**	**↓** T-cell activation in mouse spleen and T cell hybridoma, and in PBMC (Kino et al., 1987; Bierer et al., 1991)
IFN-α	**+**	↓ EC migration and proliferation in HUVEC (Friesel et al., 1987)	**+++**	Anti-viral in human blood (Neumann et al., 1998), immunomodulation in human blood (Blanco et al., 2001)
Infliximab	**+**	↓ MVD, VEGF, and EC migration in human intestinal tissue and EC (Rutella et al., 2011)	**+++**	Binds soluble and membrane TNF-α in mouse myeloma cells (Scallon et al., 2002)

*DSS, dextran sodium sulfate; HMEC-1, human microvascular endothelial cells; iKO, conditional inducible knockout; MMP-9, matrix metallopeptidase; NK, natural killer cells; PBMC, peripheral blood mononuclear cell; VEGFR2, vascular endothelial growth factor receptor 2.*

### ANTI-VEGF TREATMENT

Recently we have shown that G6-31, a monoclonal antibody that blocks VEGF, had unexpected pro-angiogenic effects, reversing the rarefied peripheral lung microvasculature and restoring the tissue TSP-1 and Ang-2 levels in *Eng*^+/-^ and *Alk1^+/^*^-^ mouse models of HHT, respectively (Ardelean et al., 2014a; **Table 1**).

Several clinical reports noted that HHT patients treated systemically with 5-10 mg/kg of bevacizumab, a humanized anti-VEGF antibody, showed a significant improvement in the frequency of epistaxis, number of required blood transfusions and that some patients no longer needed a liver transplantation (Mitchell et al., 2008; Bose et al., 2009; Oosting et al., 2009; Retornaz et al., 2009). These data were confirmed in a phase 2 clinical trial that included HHT patients with severe liver involvement (Dupuis-Girod et al., 2012). Interestingly, five of the eight patients treated in this trial with the anti-VEGF therapy also showed alleviation of secondary PAH. However, the effects of anti-VEGF therapy on lung and brain AVMs have not been studied to date in HHT patients.

Other studies demonstrated that intranasal topical or submucosal injections of bevacizumab, at 25–100 mg/dose, decreased the frequency of epistaxis in several HHT patients (Simonds et al., 2009; Chen et al., 2011). Yet, the mechanisms through which bevacizumab exerted these effects are poorly understood.

Despite these promising results, anti-VEGF treatment, especially at high dose and/or administered for long-term, can have side effects including hypertension, severe proteinuria, gastrointestinal bleeding, and wound dehiscence (Pavlidis and Pavlidis, 2013). These effects are reversible and possibly avoided with close monitoring, lower doses and shorter regimens of anti-VEGF therapy. For example, systemic bevacizumab at 1 mg/kg showed promising results by reducing nasal and gastrointestinal bleeding in a patient with HHT (Suppressa et al., 2011). Moreover, preliminary results of a phase-1 double-blind single center study, evaluating the safety of a bevacizumab nasal spray in HHT-associated epistaxis, suggested no outcome differences between low (12.5 mg), intermediate (50 and 75 mg), or high (100 mg) doses (Dupuis-Girod et al., 2014). Therefore, lower intranasal anti-VEGF doses may be the preferred therapeutic approach in reducing the nosebleeds in HHT. Overall, off-label use of anti-VEGF therapy showed promising results in HHT. However, caution is warranted, as the mechanisms of AVM formation in HHT are yet to be determined and the effects of this therapy, particularly on lung and brain AVMs, are still unknown.

### THALIDOMIDE

Thalidomide and the second-generation analog lenalidomide, are medications with anti-angiogenic and immunomodulatory effects (**Table 1**), used clinically in multiple myeloma and erythema nodosum of leprosy (Teo, 2005). The anti-angiogenic effects of thalidomide are dose-dependent (**Table 1**).

In HHT patients, thalidomide given orally at 50–200 mg/day decreased the number of telangiectases, epistaxis and transfusions (Chen et al., 2012; Franchini et al., 2013). However, thalidomide doses ≥100 mg per day can cause peripheral neuropathy, somnolence, constipation and deep vein thrombosis. The side effects of thalidomide can be attenuated by reducing the dose given (Teo, 2005).

In several HHT patients, oral lenalidomide at 10–15 mg/day, reduced the number of gastrointestinal bleeds (Bowcock and Patrick, 2009). Lenalidomide inhibited VEGF production and migration of EC, hence showed anti-angiogenic properties, and had improved immunomodulatory effects and safety profile, when compared to thalidomide. The most common side effects of lenalidomide were anemia, constipation and drowsiness (Teo, 2005).

### PROPRANOLOL AND TIMOLOL

Propranolol and timolol are non-selective beta-adrenergic receptor (β-AR) blockers with anti-angiogenic properties (**Table 1**). Both medications are vasoconstrictive by inhibiting the effects of adrenaline on the endothelial β-AR. Propranolol showed anti-proliferative and apoptotic effects on human umbilical vein endothelial cells (HUVEC; Xie et al., 2013), hence, has anti-angiogenic properties (**Table 1**). In addition, propranolol reduced VEGF and MMP-9 tissue expression (Storch and Hoeger, 2010), thus indirectly inhibiting angiogenesis. Interestingly, propranolol diminished *in vitro* endoglin and ACVRL1 mRNA and protein levels. It also decreased PAI-1 levels, a downstream protein of the TGF-β1-ALK5-endoglin-mediated signaling pathway, effect that can increase bleeding in HHT (Albinana et al., 2012). Clinical data to confirm these potentially undesirable effects in HHT are lacking. One case report indicated that intranasal timolol (0.5% ophthalmic solution) reduced the frequency and severity of epistaxis in one HHT patient, after 3–4 days of treatment (Olitsky, 2012). The results of a clinical trial testing the effect of topical timolol on cutaneous telangiectases in HHT are pending (http://clinicaltrials.gov/show/NCT01752049).

### TACROLIMUS (FK506, PROGRAF)

Tacrolimus is an immunomodulator macrolide with anti-angiogenic properties (**Table 1**), used in organ transplantation (Posadas Salas and Srinivas, 2014), some patients with rheumatoid arthritis, lupus nephritis (Tanaka et al., 2012), ulcerative colitis (Inoue et al., 2013) and topically, in severe eczema and vitiligo.

One case report noted that a woman diagnosed with HHT, hepatic AVMs and high-output cardiac failure, treated in the first year post liver transplantation with sirolimus (rapamycin), an inhibitor of mTOR, low dose tacrolimus (trough levels 2–5 ng/ml/day), prednisone (5 mg/day in the first 6 months) and aspirin (81 mg/day), experienced cessation of mucosal bleeding, normalization of hemoglobin levels and disappearance of cutaneous and gastrointestinal telangiectases. This suggests that the combined immunosuppressive therapy might have exerted anti-angiogenic effects on telangiectases (Skaro et al., 2006). Patients treated with tacrolimus need to be monitored for potential adverse effects, such as severe infections, hypertension, renal and central nervous system dysfunction.

### INTERFERON-α

Interferon-α is a cytokine with anti-viral and immunoregulatory properties, used to treat some patients with chronic hepatitis and certain hematological cancers (Platanias, 2005). IFN-α binds to type I IFN receptors, inducing their phosphorylation, stimulation of the transcription factors Stat1 and Stat2, and activation of multiple target genes. However, it is less known that IFN-α also has some anti-angiogenic properties (Friesel et al., 1987; **Table 1**).

In two patients with HHT and co-morbidities, metastatic renal cancer and chronic hepatitis C respectively, INF-α (3 million units injected subcutaneously three times a week for 3 and 30 months respectively) had beneficial effects on epistaxis and telangiectases (Massoud et al., 2004; Wheatley-Price et al., 2005). Potential side effects of IFN-α include decrease in blood cell count, and liver and thyroid dysfunction.

### INFLIXIMAB (REMICADE)

Infliximab is a chimeric anti-tumor necrosis factor (TNF)-α monoclonal antibody that binds soluble and membrane-bound TNF-α. Thus, it is used to treat rheumatic and inflammatory bowel diseases (IBD; Willrich et al., 2014). Recent *in vivo* and in *vitro* data from patients diagnosed with IBD and treated with infliximab indicated that this anti-TNF-α antibody had some anti-angiogenic properties (Rutella et al., 2011; **Table 1**).

Interestingly, infliximab (5 mg/kg intravenously administered every 8 weeks for a total of 40 infusions) given to a 21 year-old man with refractory Crohn’s disease and HHT, on chronic iron infusions, reduced the frequency of nosebleeds and stabilized the hemoglobin levels after the third dose (Papa et al., 2010). As HHT is a non-inflammatory vasculopathy, this observation supports the concept that infliximab might have intrinsic anti-angiogenic properties that could be beneficial in HHT. Given that infliximab is an immunomodulatory medication, several adverse effects can occur: severe infections, demyelinating diseases and potentially lymphomas (Willrich et al., 2014).

## CONCLUSION

Dysregulated angiogenesis, induced by a tissue imbalance in angiogenic and angiostatic factors, plays a role in the pathogenesis of HHT. However, the mechanisms that predispose to and are responsible for AVM formation in HHT are yet to be discovered. Surprisingly, VEGF blockage led to increased MVD and restoration of the TSP-1 and Ang-2 balance in the lungs of *Eng^+/-^* and *Alk1^+/-^* mice respectively, and prevented or decreased AVM formation in the *Alk1* iKO^e^ mice. Hence, even though VEGF is not a major pathogenic factor, it acts as a critical modulator of angiogenesis in HHT.

Drugs that target directly or indirectly the angiogenic vessels and the master angiogenic regulator VEGF, showed promising results in HHT. However, caution is warranted, as systemic anti-angiogenic treatment can also alter the vasculature in the unaffected organs. The potential side effects of systemic anti-angiogenic therapy are generally reversible with discontinuation of treatment. Moreover, the effects of anti-VEGF therapy on human AVMs are currently unknown. Several immunomodulatory agents display some anti-angiogenic properties. In addition, immune modulation, possibly through regulation of common immune and angiogenic pathways, may be an option for patients with HHT and co-morbidities of immune dysregulation. Overall, anti-angiogenic therapies showed some encouraging results in HHT. However, more mechanistic studies and larger clinical trials are required to fully understand the implications of using anti-angiogenic therapies in HHT.

## Conflict of Interest Statement

The authors declare that the research was conducted in the absence of any commercial or financial relationships that could be construed as a potential conflict of interest.
